# Reacción granulomatosa de tipo sarcoideo secundaria a biopolímeros: reporte de caso y revisión de la literatura

**DOI:** 10.7705/biomedica.6059

**Published:** 2022-05-01

**Authors:** María Fernanda Corrales, Rodrigo Restrepo, Estefanía Calle, Daniela Morales

**Affiliations:** 1 Grupo de Investigación, Universidad CES, Medellin, Colombia Universidad CES Universidad CES Medellin Colombia

**Keywords:** granuloma de cuerpo extraño, biopolímeros, sarcoidosis., Granuloma, foreign body, biopolymers, sarcoidosis.

## Abstract

La alogenosis iatrogénica es la enfermedad causada por la aplicación de biopolímeros con fines estéticos. Sus manifestaciones clínicas pueden presentarse entre las seis horas y los 30 años posteriores a la aplicación, con síntomas locales o sistémicos. El principal rasgo de la histopatología es la presencia de granulomas por cuerpo extraño con reacción de tipo sarcoideo, la cual es difícil de Interpretar por su asociación con la sarcoidosis. Se reporta aquí el caso de una paciente con lesiones granulomatosas de tipo sarcoideo por reacción a cuerpo extraño, secundaria a la aplicación de múltiples sustancias desconocidas en cara y glúteos.

## Abstract (en)

Iatrogenic allogenosis is a disease caused by the injection of biopolymers with esthetic purposes. Clinical manifestations can occur between six hours and 30 years after the procedure with local and/or systemic symptoms. The pathological findings are characterized by the presence of foreign body granulomas with a sarcoid-like reaction. Its Interpretation Is difficult given Its association with sarcoidosis. We report the clinical case of a female patient with granulomatous lesions In reaction to a foreign body secondary to the multiple application of unknown substances on the face and buttocks.

Durante años se han explorado materiales en busca de una sustancia que no sea alergénica, carcinogénica, ni inflamatoria, que no produzca reacciones a cuerpo extraño y proporcione volumen y contorno en diversas áreas del cuerpo según la necesidad y deseo del paciente. Sin embargo, los materiales utilizados, como la silicona líquida, el colágeno, el metilmetacrilato y el gel de poliacrilamida, entre otros, se han asociado con numerosas complicaciones.

En este contexto, las reacciones autoinmunitarias producen cambios histológicos que conducen a la aparición, en las etapas iniciales, de macrófagos con material oleoso en el citoplasma y, cuando la inflamación es crónica, a la formación de granulomas ^1^. Los granulomas secundarios a cuerpo extraño se manifiestan clínicamente como induraciones, nódulos e irregularidades que pueden ser la única manifestación. No obstante, el espectro clínico del término “alogenosis iatrogénica” incluye un gran número de manifestaciones, tanto locales como sistémicas, que pueden producir desde cambios leves hasta deformidad y falla multiorgánica.

## Caso clínico

Se reporta el caso de una mujer de 50 años, previamente sana, que consultó inicialmente con un cuadro clínico de un año de evolución consistente en la aparición de lesiones nodulares dolorosas en párpados, frente y surcos nasogenianos, las cuales fueron aumentando progresivamente de tamaño hasta convertirse en una pequeña úlcera en la región temporal derecha que drenaba un material viscoso. La paciente refería, además, la aparición de otros nódulos más pequeños, asintomáticos, en las mejillas, el cuello y el dorso de las manos, que habían cambiado en los últimos meses.

Relataba, asimismo, que hacía más de diez años le había sido inyectado material de relleno para mejorar las líneas de expresión de los párpados, el entrecejo, la frente y los pliegues nasolabiales. La paciente desconocía el producto que le habían aplicado y ya había sido tratada con prednisolona y siete ciclos de ciclofosfamida intravenosa ante la sospecha de sarcoidosis relacionada con la aplicación del material de relleno, pero no había habido mejoría clínica con estas terapias.

En el momento de la evaluación, refería que su calidad de vida y su autoestima se veían muy afectadas, lo que la había llevado al aislamiento social y a tratar de ocultar sus lesiones con grandes lentes de sol y abundante maquillaje.

**Figura 1:**
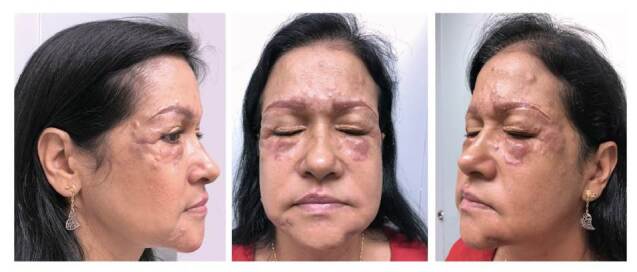
Múltiples placas y nódulos eritematosos, bien definidos, indurados y adheridos a planos profundos, los cuales estaban localizados en las regiones frontal, periorbitaria, interciliar, temporal y malar, y los surcos nasogenianos

En el examen físico presentaba múltiples placas y nódulos eritematosos, bien definidos, indurados y adheridos a planos profundos, localizados en las regiones frontal, periorbitaria, interciliar, temporal y malar, y en los surcos nasogenianos. Llamaba la atención una úlcera de 0,5 cm con costra hemática superficial en la región temporal derecha que, al ser presionada, drenaba un líquido cetrino de consistencia oleosa. También, se observaban cientos de nódulos normocrómicos, subcutáneos, menores de 1 cm en mejillas, cuello, pecho, antebrazos y el dorso de ambas manos (figura 1).

Se le practicó una resonancia magnética con contraste de tejidos blandos en cara y cuello, en la cual se evidenció un proceso inflamatorio con presencia de nódulos y reacción a cuerpo extraño en el tejido celular subcutáneo en las áreas cigomática, orbitaria e infrapalpebral, y en las dos bolsas de Bichat. En el pilar amigdalino izquierdo, se encontró engrasamiento y un nódulo de 8 mm de diámetro, así como múltiples ganglios sublinguales y en el mediastino superior, menores de 5 mm.

En la ecografía de tejidos blandos, se encontraron en la cara múltiples lesiones pseudonodulares en “tormenta de nieve” dispersas en las regiones frontal, periorbitaria y malar, de predominio derecho, segestivas de inyección de agentes exógenos.

La biopsia de piel evidenció epidermis ortoqueratósica de grosor normal sin cambios significativos. En la dermis papilar y reticular media y profunda, se encontraron múltiples granulomas desnudos conformados por histiocitos epitelioides con escasos linfocitos de aspecto maduro y células gigantes multinucleadas del tipo de cuerpo extraño. Se reconocieron, además, histiocitos macrovacuolados y microvacuolados y algunos cuerpos asteroides.

**Figura 2:**
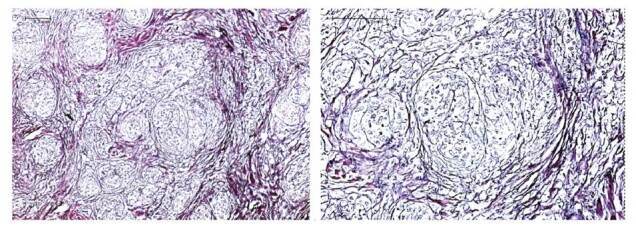
Hallazgos de reacción granulomatosa a cuerpo extraño (material de relleno) de tipo sarcoideo

La coloración del retículo resaltó la reticulina alrededor de los granulomas y, ocasionalmente, en su interior. Además, las tinciones de Ziehl-Neelsen, PAS y plata metenemina, con controles positivos adecuados, fueron negativas para hongos y bacilos ácido-alcohol resistentes. Los hallazgos, sin ser diagnósticos, son los de una reacción granulomatosa a cuerpo extraño (material de relleno), de tipo sarcoideo (figura 2).

Además, para descartar la sarcoidosis sistémica, se hicieron estudios con tomografía computarizada de pulmones y pruebas para determinar los niveles de la enzima convertidora de angiotensina, la 1,25 dihidroxivitamina D, el calcio sérico y el calcio urinario en 24 horas, los cuales se encontraron dentro de los límites normales. Con base en los hallazgos, la impresión diagnóstica fue la de una alogenosis o reacción granulomatosa a cuerpo extraño que simulaba una sarcoidosis cutánea localizada.

Se inició el tratamiento con 200 mg de hidroxicloroquina cada 12 horas, 0,5 mg de colchicina cada 12 horas y tacrolimus al 0,1 % aplicado en ungüento tópico en la noche. Tres meses más tarde, la paciente mostraba una excelente tolerancia a los medicamentos, habían desaparecido completamente los nódulos del cuello y las extremidades superiores, y el eritema y el dolor de las lesiones en la cara habían disminuido. Sin embargo, continuaba muy insatisfecha con su aspecto físico, por lo que se decidió adicionar 50 mg/día de talidomida al tratamiento.

Con la talidomida, la paciente presentó una mejoría clínica muy significativa, pero tuvo que ser suspendida cuatro meses después de su inicio por la aparición de una neuropatía periférica sensitiva confirmada en la electromiografía de control, síntoma que no aparecía en el estudio basal. La neuropatía se resolvió completamente al suspender el tratamiento y no hubo empeoramiento de las lesiones en la piel.

**Figura 3:**
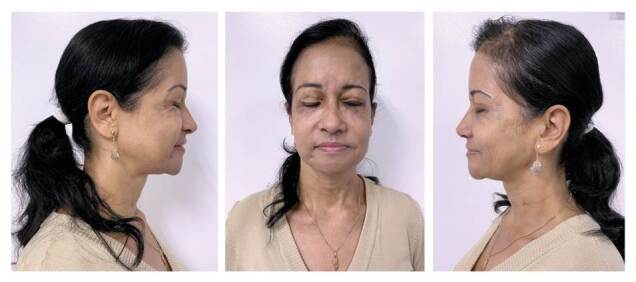
Mejoría marcada de las lesiones después del tratamiento médico y quirúrgico

Después de meses de estabilidad clínica con la hidroxicloroquina y la colchicina, se procedió a la extracción quirúrgica de los nódulos restantes, a cargo de especialistas en oftalmología oculoplástica y cirugía plástica, con excelentes resultados cosméticos y recuperación de la autoestima de la paciente (figura 3).

## Consideraciones éticas

La paciente firmó el consentimiento informado autorizando el uso de sus datos y de sus fotografías con fines académicos.

## Discusión

La alogenosis iatrogénica, término acuñado por el doctor Felipe Coiffman en el 2008, comprende los efectos secundarios a la aplicación de biopolímeros o sustancias alógenas por el personal de la salud ^2^. La mayoría de las complicaciones por el uso de estas sustancias se debe a su aplicación por parte de personal no médico y, también, se la ha denominada alogenosis secundaria, enfermedad por sustancias modeladoras, enfermedad por sustancias de relleno o enfermedad por adyuvantes. En la actualidad, la alogenosis es una condición reconocida en todo el mundo, especialmente en Latinoamérica, en donde es clara la ausencia de control sanitario de los productos que se aplican y del personal que practica los procedimientos ^3^.

La popularidad de los procedimientos estéticos, especialmente cuando son de bajo costo, ha provocado un aumento exponencial en el número de complicaciones secundarias al uso de sustancias para moldear y de relleno, precipitando un extenso problema de salud pública en Colombia, a pesar del subdiagnóstico, aunque es evidente el incremento de la incidencia de complicaciones.

Los biopolímeros son moléculas derivadas del petróleo, de origen vegetal o sintético ^4^. Antes se creía que eran productos inertes que permitían el moldeamiento de la cara y el cuerpo según el deseo y la necesidad de cada paciente. Sin embargo, con el transcurso del tiempo, se ha demostrado que estas sustancias no son inocuas y originan complicaciones a corto y a largo plazo, incluidas infecciones, reacciones a cuerpo extraño, deformidad física, alteraciones orgánicas y enfermedades autoinmunitarias ^5^.

El primer reporte de complicaciones secundarias a la aplicación de estas sustancias lo presentó Balzer en 1886, quien describió la presencia de induraciones cutáneas producidas por sustancias oleosas. Otros autores, como Mook y Wander, describieron en 1920 la presencia de tumores blandos en pacientes a los que se les habían aplicado inyecciones de aceite de alcanfor y, en 1928, Woringer hizo referencia a granulomas como reacción a cuerpo extraño. Actualmente, México es el país que más publica sobre las complicaciones de esta enfermedad y el Hospital General de México se ha convertido en centro de referencia en la atención de los pacientes que la presentan ^6^.

Ante la presencia de las sustancias empleadas para moldear, el cuerpo presenta una reacción inflamatoria aguda y una crónica. La reacción inflamatoria inicial se produce en el intento de encapsular el material extraño, induciendo cambios fenotípicos de las células fagocíticas, lo que lleva a su transformación en células gigantes multinucleadas ^5^. La fase aguda se caracteriza por la migración de neutrófilos y la presencia de exudado proteico, en tanto que, en la fase crónica, aparecen cúmulos de linfocitos y monocitos que finalmente se diferencian en macrófagos, produciendo la liberación de citocinas e interferón gama (INF-gamma) y alfa (INF-alfa), lo que resulta en la formación de granulomas ^7^.

Con el depósito de material antigénico en el tejido, se inicia la formación del granuloma; las células mononucleares presentadoras de antígenos fagocitan dicho material por endocitosis y las proteínas antigénicas son degradadas por proteasas en los lisosomas para ser presentadas por el complejo mayor de histocompatibilidad II a los linfocitos T CD4+, lo cual desencadena una respuesta Th1, con producción de INF-gama, o unaTh2 con producción de IL- 4, IL-5 o IL-13, dependiendo de la naturaleza de la sustancia inyectada ^7^.

Dado el espectro de la alogenosis iatrogénica, se cree que las manifestaciones clínicas del paciente son consecuencia del estado inmunológico previo; se producen cuadros clínicos leves y otros graves, con periodos de reagudización desencadenados por traumas, infecciones, u otras sustancias o antígenos que llevan a la activación del sistema inmunitario y a la pérdida de la tolerancia inmunológica y el reconocimiento de antígenos externos (como las sustancias de relleno), o de antígenos propios, produciendo así la enfermedad reumática. Se considera que los pacientes con antecedentes familiares reumatológicos tienen mayor riesgo de desarrollar una reacción inmunológica exagerada, por lo que se debería evitar el uso de sustancias de relleno en ellos ^8^.

Las sustancias alógenas se han relacionado con fenómenos autoinmunitarios como esclerosis sistémica, artritis reumatoide, lupus y fibromialgia, entre otros ^5^. En el 2013, se acuñó el término “síndrome de ASIA” el cual incluye enfermedades inmunomediadas desencadenadas por un estímulo adyuvante, e incluye mialgias, artralgias, fatiga crónica, alteraciones neurológicas asociadas con la desmielinización, deterioro cognitivo, boca seca y fiebre, así como el inicio de enfermedades reumatológicas con presencia de autoanticuerpos, hallazgos histopatológicos sugestivos, y mejoría sintomática cuando se retira el agente antigénico ^9^.

El doctor Coiffman estudió 342 pacientes con alogenosis iatrogénica en Bogotá y encontró que el 95 % de ellos no sabía qué sustancia les había sido inyectada, el 70 % de las aplicaciones habían estado a cargo de cosmetólogas y, el 30 %, de médicos, incluidos dermatólogos, cirujanos plásticos, otorrinolaringólogos y odontólogos ^2^.

Según reportes, las complicaciones pueden ocurrir entre seis horas y hasta 30 años después de la aplicación y pueden ser tanto locales como sistémicas. Las manifestaciones locales incluyen dolor, eritema, pigmentación, edema, fibrosis, cicatrización queloide, infecciones, fístulas, necrosis de piel y desplazamiento del material, y las manifestaciones generales, fiebre, dolor generalizado, artralgias, malestar general, aumento de la caída del cabello, depresión y migración del material inyectado, entre otros ^2^^,^^6^. Las zonas más comúnmente afectadas son glúteos, extremidades inferiores, abdomen, párpados, región malar y labios ^10^.

Los materiales de relleno se clasifican en reabsorbibles (meses o años) y permanentes. Entre las sustancias de relleno absorbibles, se encuentran el tejido graso autólogo, el injerto dermograso, el colágeno bovino, el colágeno autólogo, el ácido poliláctico, la hidroxiapatita de calcio y el ácido hialurónico, y entre las de relleno permanentes, se encuentran el polimetilmetacrilato, la silicona, el polimetilsiloxilano, el gel de poliacrilamida, el petrolato, la vaselina y la parafina, entre otros ^11^ (cuadro 1). Como la mayoría de los pacientes desconocen qué material les fue inyectado y qué tipo en particular, se debe recurrir al apoyo histopatológico para documentar la presencia de una reacción inflamatoria a cuerpo extraño, además de estudios imagenológicos y de otros tipos, para definir su extensión y la naturaleza precisa de la sustancia inyectada.

**Cuadro 1:** Hallazgos histopatológicos según tipo de biopolímero

Sustancia	Hallazgos histopatológicos
Colágeno bovino	Masa eosinofílica, no birrefringente
Ácido hialurónico	Sustancia basófila sin estructura cuando es superficial, rara formación de granulomas
Ácido polil-L-láctico (PLLA)	Material cristaloide, birrefringente positivo, formación de granulomas de células epitelioides con células gigantes rodeadas por fibrosis
Hidrogel acrílico	Partículas poliédricas que forman granulomas tardíos, densos con células gigantes, áreas necróticas y hendiduras de colesterol
Polimetilmetacrilato (PMMA)	Partículas poligonales, irregulares, traslúcidas, descritas como vidrio roto, con poco tejido fibrótico, con pocas células gigantes que contienen cuerpos en su citoplasma
Poliacrilamida (PAAG)	Material basófilo ondulado
Silicona	Formación de granulomas con células gigantes sin partículas en su interior e infiltrados linfocíticos perivasculares
Parafina/vaselina	Células epitelioides y células gigantes asociadas con infiltrado linfocítico y fibrosis

*Basada en ^13^^,^^14^

La técnica espectroscópica de resonancia magnética permite diferenciar la estructura química de las sustancias, y proporciona información sobre el número y tipo de moléculas en una muestra. En un estudio observacional, transversal y descriptivo realizado en el Hospital General de México, se analizó la sustancia infiltrada por medio de espectrometría de resonancia magnética en 18 pacientes y se estableció el tipo de sustancia infiltrada en los tejidos. Se encontró que, en el 35 % de ellos, los granulomas fueron secundarios a la inyección de aceite comestible, en el 40 %, a aceite mineral, en el 15 %, a silicona y, en el otro 15 %, a la combinación de aceites comestibles y silicona ^12^.

En otro estudio clínico, transversal y descriptivo llevado a cabo en el mismo hospital, se utilizó una escala para evaluar las lesiones producidas por la infiltración de sustancias para moldear, la cual resultó útil para estadificar la enfermedad y determinar el pronóstico del paciente, así como para la toma de decisiones terapéuticas. Se trata de un sistema de puntuación según la cantidad de sustancia infiltrada, las zonas infiltradas, el tipo de sustancia, los síntomas y signos del paciente, las alteraciones en los resultados de laboratorio y los hallazgos en la resonancia magnética. El estadio 1 (6-10 puntos) incluye los pacientes con buen pronóstico y mejoría clínica con el tratamiento médico. El estadio 2 (11-17 puntos) incluye a aquellos con pronóstico reservado y buena mejoría clínica con el tratamiento médico, pero que pueden requerir cirugía. El estadio 3 (18-23 puntos) cubre a aquellos con pronóstico reservado que requieren manejo quirúrgico, y el estadio 4 (24-26 puntos), a aquellos con mal pronóstico, que pueden morir por falla orgánica múltiple ^13^.

Entre los diagnósticos diferenciales es importante distinguir la reacción granulomatosa generada por biopolímeros o materiales de relleno y el liposarcoma o lipoesclerosis, cuya diferenciación requiere, en ocasiones, una correlación clínica e histológica rigurosa ^14^. También, se deben tener en cuenta condiciones como la tuberculosis, la erisipela, la lepra lepromatosa, la neurofibromatosis, la esclerosis tuberosa, la amiloidosis, y la sarcoidosis, entre otras ^15^.

La sarcoidosis en una enfermedad multisistémica, con compromiso pulmonar, ganglionar, ocular, hepático, cardiaco, óseo, y del sistema nervioso central y la piel. Sin embargo, hasta el momento no tiene una definición clara por su gran variabilidad sintomática y la ausencia de métodos diagnósticos con la suficiente sensibilidad y especificidad ^16^. Se debe considerar un diagnóstico de exclusión, ya que los granulomas por cuerpo extraño pueden generar manifestaciones sistémicas e histológicas similares a las de la sarcoidosis, aunque estas no se asocian con la presencia de linfadenopatías generalizadas, esplenomegalia o compromiso oftalmológico y óseo. Hallazgos como el de lesiones que aparecen años después de la aplicación del material extraño y la detección de granulomas birrefringentes en microscopía polarizada, también favorecen el diagnóstico ^17^.

Otra dificultad del diagnóstico de los granulomas por cuerpo extraño es la descripción histopatológica del ‘tipo sarcoideo’. En algunos estudios, se ha determinado que la diferenciación entre la sarcoidosis y una reacción de tipo sarcoideo se basa en el perfil de riesgo genético e inmunológico del paciente frente al desarrollo de la sarcoidosis ^13^^,^^14^. Por lo tanto, todo reporte histopatológico de una reacción de tipo sarcoideo obliga al médico a descartar una sarcoidosis sistémica, la cual se puede presentar incluso años después de una reacción sarcoideo. Aunque no existen exámenes de laboratorio específicos que permitan descartar sarcoidosis, algunos de ellos son útiles para su diferenciación ^18^ (cuadro 2).

**Cuadro 2:** Métodos diagnósticos para sarcoidosis

Antígeno carcino embrionario^18^	Test de Kveim-Siltzbach ^19^	Gammagrafía de galio ^20^
Positivo también en diabetes mellitus, enfermedad de Gaucher, hipertiroidismo, enfermedad alcohólica grave, silicosis, histoplasmosis, beriliosis, lepra y linfangiomatosis	Inoculación intradérmica de una suspensión obtenida a partir de tejido esplénico	Positivo también en linfomas, carcinomas, tuberculosis, neumonía y silicosis
	Positivo en el 70 a 90 % de pacientes con sarcoidosis	
Permite el monitoreo de la actividad de la enfermedad y la reacción al tratamiento.	Falsos positivos de 0,7 a 2 %	Cuando se combina el ACE con la gammagrafía de galio, se aumenta la especificidad a 99 %.

Tampoco el tratamiento se encuentra establecido y solo se cuenta con tratamientos no estandarizados, la mayoría basados en reportes de caso con resultados reproducibles. Desde la perspectiva de la sarcoidosis cutánea, podemos extrapolar los resultados que se obtienen con la talidomida, medicamento que parece afectar la producción de citocinas, inhibiendo selectivamente el TNF-alfa; su acción es rápida, pero su eficacia a largo plazo se desconoce ^21^. Otras terapias reportadas incluyen corticoesteroides tópicos y orales, infiltraciones con esteroides, cloroquina, hidroxicloroquina, alopurinol, metotrexato, ¡sotretinoína, tetraciclinas, imiquimod y etanercept. Los reportes mexicanos de cirugía plástica han planteado el manejo de la enfermedad según su gravedad y la extracción quirúrgica de la sustancia infiltrada, con mejores resultados cuando esta se hace antes de que se presenten complicaciones.

En una serie de casos en el Hospital Universitario de Caracas, el Hospital Vargas de Caracas y la Clínica El Ávila, se reportaron 75 pacientes que presentaban complicaciones relacionadas con la inyección de biopolímeros. En ella se recomendó iniciar la aproximación diagnóstica con exámenes de hemoglobina libre en suero, PCR, prueba de velocidad de sedimentación globular (VSG), de anticuerpos antinucleares (ANA), de C3, C4, CH50, de anticuerpos anti-dsDNA, anti-ENA, anti-CCP, de niveles séricos de silicona, de TNF e IL-1, cultivo de secreciones (si existe) y resonancia magnética (RM) del sitio afectado. En el estudio, se sugiere iniciar el tratamiento con corticoesteroides orales de baja potencia (deflazacort), en dosis entre 12 y 15 mg por 30 a 45 días, y hacer una PCR, una prueba de VSG y una RM a los 45 días. Si los niveles de reactantes de fase aguda persisten elevados, la recomendación es administrar azatriopina, colchicina, talidomida, hidroxicloroquina o micofenolato de mofetilo como monoterapia o en combinación. Después del control de los reactantes y la RM a los tres meses, se recomienda iniciar la ciclofosfamida o la terapia biológica con anti-TNF-alfa si hay indicios de enfermedad activa ^22^.

## Conclusión

La alogenosis iatrogénica es una enfermedad cuya incidencia y prevalencia han aumentado en Colombia debido al gran número de procedimientos estéticos que se practican y la falta de regulación sanitaria de los productos y el personal encargado. Es una enfermedad compleja con consecuencias, no solo estéticas, sino económicas, productivas, psicológicas y sociales, y manifestaciones clínicas que van desde lo local a lo sistémico, acompañadas, en ocasiones, de enfermedades reumatológicas de *novo.*

Una de las mayores dificultades para su detección es la aparición de síntomas años después de la aplicación del producto, usualmente sustancias muy antigénicas con bajo grado de biocompatibilidad. Debe desplegarse un alto grado de sospecha para poder detectarla tempranamente, orientar correctamente el tratamiento y educar a los pacientes sobre las consecuencias de usar productos que no son seguros y recurrir a personal poco entrenado en este tipo de procedimientos.
